# Nurses' experience with relatives of patients receiving end‐of‐life care in nursing homes and at home: A questionnaire‐based cross‐sectional study

**DOI:** 10.1002/nop2.155

**Published:** 2018-04-19

**Authors:** Bård Paulsen, Roar Johnsen, Hans Hadders

**Affiliations:** ^1^ Health service research SINTEF Technology and Society Trondheim Norway; ^2^ Department of Public Health and Nursing Norwegian University of Science and Technology (NTNU) Trondheim Norway

**Keywords:** awareness of dying, end‐of‐life care, home‐based care, nursing, nursing homes, relatives

## Abstract

**Aim:**

The aim of this study was to explore any differences between nurses working in nursing home and home‐based care in their experiences regarding relatives' ability to accept the imminence of death and relatives' ability to reach agreement when deciding on behalf of patients unable to consent.

**Design:**

An electronic questionnaire‐based cross‐sectional study.

**Method:**

An electronically distributed survey to 884 nurses in long‐term care in Norway in May 2014. A total of 399 nurses responded (45%), of which 197 worked in nursing homes and 202 in home‐based care.

**Results:**

Nurses in home‐based care, more often than their colleagues in nursing homes, experienced that relatives had difficulties in accepting that patients were dying. Nurses who often felt insecure about whether life extension was in consistency with patients' wishes and nurses who talked most about life‐prolonging medical treatment in communication with relatives more often experienced that relatives being reluctant to accept a poor prognosis and disagreements between relatives in their role as proxy decision makers for the patient.

## 1 INTRODUCTION

The Norwegian healthcare system is based on the Scandinavian public welfare model and mainly financed by the income tax system. Health care is a legislative right for all, whether specialized care in hospitals or long‐term care in nursing homes or at home. Specialized care is financed and regionally organized by central state health authorities. Primary health care, including long‐term care, is financed and organized by the municipalities.

This study was part of a larger study: *Utilization of health care services at the end of life*, which aimed to explore structural and individual factors influencing end‐of‐life care implementation and use in Norway, before and in the wake of the *Coordination Reform* (Kalseth & Theisen, 2017). Like most European countries, Norway is facing a rapid growth of older people in the population. This will increase the demand for health care, both in hospitals and in municipality‐based long‐term care. As a part of a strategy to prepare the healthcare system to meet these demands, the Norwegian Government launched the *Coordination Reform* in 2012 (HOD, 2009). As the name implies, a central aim of this reform was to improve coordination between various healthcare providers. The explicit aim of this reform was to reduce pressure on specialized healthcare providers, by transferring to municipalities all kinds of health services that did not necessarily have to take place at specialized level. A central element of this policy was increased decentralization. This was achieved by discharging patients from hospitals to municipal care as soon as possible. Consequently, the demand for long‐term care in municipalities increased substantially. During 2012, when the *Coordination Reform* was implemented, approximately 48% of all deaths in Norway took place in nursing homes and 15% in patients' own homes. “Nursing homes” include all residential institutional deaths not included in the hospital category (Statistics Norway, 2013). In Norway, relatively few patients die at home, compared with, for instance, Denmark, Sweden, United Kingdom and Ireland (Kalseth & Theisen, 2017; Official Norwegian Report, 2017). In their recent publication *Trends in place of death: The role of demographic and epidemiological shifts in end‐of‐life care policy* (2017), Kalseth and Theisen underscore that where people die reflects the organization of end‐of‐life (EOL) care, as well as major demographic and epidemiological trends. Using data on all deaths in Norway for the period 1987–2011, they found a 16% increase in nursing home deaths and a 4% decrease in deaths at home (Kalseth & Theisen, 2017). Kalseth and Theisen's data confirm that the decentralizing trend in place of death was established well before the launch of the *Coordination Reform*. However, in the period 1987–2011 then, proportion of older people was remarkably stable. This stable trend reflects the low birth rates during the thirties and during the second World War. The post‐World War “baby boom” creates a rapid growth of older people and increases the pressure significantly on long‐term care in the municipalities (Mørk, 2011).

In Norway, increased demand for long‐term care in municipalities increase the demand for health‐care professionals, nurses and physicians, who are competent in EOL care. There are some major differences in the organization of EOL care between nursing homes and home‐based care in Norway. Among other things, the clinical experience of healthcare personnel over time makes a difference and that collegial support at nursing homes is much stronger than in home‐based care setting, where nurses more often operate alone. Contemporary palliative care in Norway includes a wide spectrum of diseases, in addition to a focus on oncology. Due to this, nurses dealing with EOL care in nursing homes and in home‐based care face an increasing challenge (Official Norwegian Report, 2017).

Nursing homes usually have an attached physician on a part‐time basis, usually a general practitioner (GP). In home‐based care, nurses collaborate with the patient's GP. In a recent national evaluation of palliative care in Norway by Melby and team, however, one of the conclusions were that involvement of both family physicians and nursing home physicians often was insufficient (Melby, Das, Halvorsen, & Steihaug, 2016). Every day, nurses are the ones who have most contact with the patient and the relatives involved.

## 2 PALLIATIVE CARE

The establishment of St Christopher's Hospice in London in 1967, by Cicely Saunders and her colleagues, can be seen as a turning point in the development of modern hospices and palliative care (Clark, 1998). The application of palliative care entails a clinical shift from cure to comfort. Central concerns within the hospice movement and palliative care are to control symptoms and to provide patients with an encompassing care, which also includes relatives. Palliative care and medicine is a discipline, which emerged as a logical extension and further development of the ideas within the hospice movement (Clark & Seymour, 1999; McNamara, 2001; Official Norwegian Report, 2017). The ideology of so‐called good death or dignified death has served as a central philosophical concern and standard within the hospice movement and palliative care. However, this ideology has been contested (McNamara, 2001, pp. 45–53; Timmermans, 2005). Communication is a key concept. One of the elements of a “good death” is the fostering of an “open awareness” of imminence of death through communication, where the dying person is supported by family and friends (Clark, 2002, p. 907).

## 3 RELATIVES' DILEMMA: OPEN AWARENESS OR NOT?

As outlined above, dying patients' and their relatives' awareness of the imminence of death has been an important issue in EOL care (Borneman, Irish, Sidhu, Koczywas, & Cristea, 2014; Glaser & Strauss, 1965; Richards, Ingleton, Gardiner, & Gott, 2013; Small & Gott, 2012; Timmermans, 1994). Glaser and Strauss's term *open awareness* characterizes a situation where the imminence of death is accepted and openly talked about in communication between the patient, the family and health‐care personnel (Glaser & Strauss, 1965; Timmermans, 1994). In their review of the contemporary relevance of Glaser and Strauss studies about dying in hospitals in California Bay area in the 1960s, Small and Gott underline that Glaser and Strauss was avowedly reformist, in favour of more open awareness and improved EOL care.

In recent years, open awareness is considered crucial in modern EOL care, palliative care ideology in Norway, and is an important part of *National action program with guidelines for palliation in oncology* (HOD, 2010; Official Norwegian Report, 2017). Open communication among patients, their relatives and health‐care professionals is critical to accommodate and support relatives (Hadders, Paulsen, & Fougner, 2013; Loke, Quiping, & Leung, 2013; Masson, 2002; Mossin & Landmark, 2011). Nevertheless, health‐care personnel often are reluctant to give conclusive information of the imminence of death to the patient and the patient's relatives. If they do, however, there are several possible reasons why relatives struggle to face clinical information about impending death (Biola et al., 2007; Curtis et al., 2005; Melby et al., 2016). At the bedside of a terminally ill patient, relatives are often unprepared for what they observe and experience even if they receive clear information about patient's terminal condition. Realizing that there is no hope for recovery is very difficult (Meeker & Jezewski, 2008).

Observing their loved one's suffering, not knowing what to do about it lead to feelings of helplessness and uselessness (Sand & Strang, 2006). Relatives who are unable to accept the patient's end of life may insist on medical interventions, which are both useless and counterproductive for the patient. In a Norwegian study, Dreyer and colleagues found that relatives sometimes tried hard to force a dying patient to eat and drink, even when the patient clearly expressed that this was unwelcome (Dreyer, Førde, & Nortvedt, 2009). Relatives, then, need repeated information and help to understand what is going on and what to expect.

EOL care confronts nurses with great challenges as they spend much time with the individual patient and her relatives and they meet people in a situation of fear, despair and anxiety (Melin‐Johansson, Henoch, Strang, & Browall, 2012). In a study by Kenneth White and colleagues, oncology nurses were asked to list skills considered important in their work. “How to talk to patients and their relatives about dying” was ranked as the most important one (White, Coyne, & Patel, 2001). There seems, then to be a growing need for more knowledge and reflection on the difficult and complex interplay between the dying patient, the relatives and the nurses in their role as bedside helpers and supporters in EOL care in municipalities in Norway (Hadders et al., 2013; Hofstad, 2017; Melby et al., 2016).

### 3.1 The relatives' role as proxy decision makers

In Norway *The law on patient rights* (*Pasientrettighetsloven*) specifies who is to be considered next of kin (*§ 1–3*) and specifies the rights a patient's relatives have to obtain information about treatment in the clinic as follows (*§ 3–3*): “When patients consent and when conditions vouch for it, the patient's nearest relatives shall receive information about the patient's health and the treatment given” (HOD, 1999). Next of kin, in this context, is anyone within his or her social network who the patient defines as a “significant other.” If the patient is cognitively impaired and unable to consent to important decisions about care, relatives are legally entitled to act as proxy decision makers on behalf of the patient (HOD, 1999). In this situation, relatives could be confronted with difficult questions about whether or not to try to prolong the dying patient's life, or let the suffering end (Romøren, Pedersen, & Førde, 2016). Making these kinds of decisions on behalf of their loved one can be a heavy burden for the patient's relatives, leaving them feeling unprepared and helpless (Dreyer et al., 2009). They usually feel unqualified and ill prepared to decide on these matters and may feel guilty. “They could be exhausted, they questioned their own motives and afterwards they had a bad conscience because they felt they had considered their own feelings more than those of their dying relative” (Dreyer et al., 2009, pp. 675–6). A family member, who takes on the role of decision maker and are met with criticism, may face severe emotional and existential problems (Elliot & Olver, 2005; Forbes, Bern‐Klug, & Gessert, 2000).

Studies of the interplay between patients, their relatives and health‐care personnel supporting and caring for them often conclude that the quality of the information healthcare professionals provide in such situations is decisive (Carlson, 2007; Hadders, 2007). The need for relevant, thorough and timely intervention and information is a focal point in professional guidelines and plans for EOL care. Liverpool Care Pathway (LCP) is such a tool that has been used in several countries, including Norway. LCP was originally developed in Great Britain during 1990s to avoid unnecessary treatment and facilitate a palliative care focus during the three last days of patients with cancer. Later, it has been applied for patients with a variety of diagnoses in EOL care (Melby et al., 2016). However, in Great Britain, LCP has been criticized and abandoned in favour of other tools, partly due to overly standardized rigid use by healthcare professional with lacking competence (Melby et al., 2016, p. 28). In Great Britain, *National Institute for health and Care excellence* (NICE) have developed a new revised version of LCP. A version of LCP has been adopted in Norwegian EOL care over the last decade and in sporadic use in various parts of Norway. By 11th March 2017, 326 nursing homes and 96 home‐based care facilities used Norwegian version of LCP (Official Norwegian Report, 2017, p. 57). However, this plan has not been applied as a national standard in EOL care in Norway (Melby et al., 2016).

### 3.2 Nurses' double objective: helping relatives to take care of the patient

In their work with dying patients, health‐care professionals must care for both the dying patients and their relatives (Benzein, Hagberg, & Saveman, 2008). Relatives need a realistic understanding of the patient's condition and prognosis to be able to accept the situation and help their loved one in the best way. Bewildered relatives, denying imminent death and clinging to medically unrealistic hopes may add to the physical and psychical suffering of the patient. Further, if a patient is unable to consent, the relatives need time to prepare for their role as proxy decision makers on behalf of the patient. The aim of this study was to explore any differences between nurses working in nursing home and home‐based care in their experiences regarding relatives' ability to accept the imminence of death and relatives' ability to reach agreement when deciding on behalf of patients unable to consent.

## 4 MATERIAL AND METHOD

To construct a questionnaire, customized to Norwegian healthcare system and suitable to electronic distribution to nurses working in home‐based care and in nursing homes, we conducted semi‐structured interviews with seven nurses in home‐based care and nursing homes. How to cooperate with a dying patient's relatives was a central theme in all the interviews. Two main challenges in the cooperation with the relatives emerged: How to deal with lack of acceptance of the imminence of death and how to handle disagreement between the relatives when they should act as proxy decision maker for patients unable to consent. We, also, interviewed four relatives of recently deceased patients where we thematically focused on their considerations of how patients and their relatives were taken care of. Interviews were transcribed in full length. The authors used this material to obtain a broad understanding of EOL care in various organizational settings and to single out important topics confounding any differences between care in nursing homes and home‐based care. In addition, the transcribed dialogues with nurses were used as a source of examples of formulations and wordings used by nurses, to strengthen the relevance of the questionnaire for our respondents.

The authors developed the questionnaire together through a process of discussion and revision and ended up with 65 questions. The two main questions on cooperation with relatives were constructed as: “Does it happen that the patient's relatives, in spite of the information you give them, find it difficult to accept that the patient is dying?” and the other regarding the degree to which disagreements occurred when relatives were acting as proxy decision makers for patients unable to consent: “Does it happen that disagreement occur between relatives in these situations?” Response alternatives for both questions were “Quite often,” “Sometimes,” “Seldom” and “Never.”

All 65 questions were organized in four sections. Section 1 was named “Background,” containing questions concerning the individual respondent; age, years of service in end‐of‐life care, place of work (nursing home, department for home‐based care) and some data about the municipality where the respondent worked. Section 2 was named “Working with dying patients in your department,” with the aim to obtain data characterizing usual practice regarding EOL care in nursing homes and departments of home‐based care where the individual respondents worked. Important care topics in this section were organization of EOL care and communication with relatives and patients. In sections 3 and 4, care‐related collaborations with other parts of the municipal health‐care organization and with nearby hospitals were the main topics. Questions concerning practice of work were concrete. However, “common practice” usually has exceptions. Questions regarding regular practice had to be formulated accordingly, using wordings like, for instance: “Do you usually …” with alternatives on the formula “Always,” “Usually,” “Usually not.” Alternatively, questions had the form “Does it happen that …,” with “Quite often,” “Sometimes,” “Very seldom” and “Never” as alternatives. To measure the importance of various topics in the communication between nurses and patients' relatives, a list of relevant topics was constructed. To the question; “From your experience: how much time do you use on various topics in communication with relatives?” respondents were offered the alternatives: “A lot,” “Some,” “Little” regarding each of the topics in question.

Prior to the survey, the questionnaire was circulated, discussed among members of a group of researchers associated to the project and validated by three nurses, of whom one worked in a nursing home and two in home‐based care. One of these two represented a small and remote municipality, the other a large city. Having completed the questionnaire, these nurses were asked to give their opinions on relevance of the questions for their work with dying patients and the appropriateness of formulation of questions and alternatives for answers. Revisions were made according to nurses' response and the questionnaire was prepared for electronic distribution.

Introduction letters and surveys were sent to the health administration departments in every Norwegian municipality with a request to distribute questionnaires to two experienced nurses, one in a nursing home and another in home‐based care. The wording “experienced nurses” was used to recruit nurses familiar with long‐term care in either nursing homes or in home‐based care. No further specifications were made, regarding, for example, specialized education or years of service in long‐term care. In Oslo, the capital city of Norway, requests were sent to each of 15 city district administrations. Requests were sent to a total of 442 municipal health administrations, including the 15 Oslo city districts, covering all municipalities in Norway. Three weeks later, a reminder was posted to all non‐responders. A total of 399 nurse responded, 197 working in nursing homes and 202 in home‐based care, representing 45% and 46% of municipalities, respectively.

### 4.1 Ethical considerations

The Regional Committee for Medical and Health Research Ethics has approved the study, approval number 2012/852. Oral consent was sought in all interviews.

## 5 ANALYSIS

*SPSS_Statistics_Win 64* was applied for data analysis. Binary logistic regression was used to test associations between the two outcome variables; the degree to which nurse's experience “Relatives not accepting that death is close” and “Disagreements among relatives concerning life prolongation” and variables characterizing variations regarding nurses' communication with patients' relatives, organization of the care, personal characteristics of the nurse herself and characteristics of the municipality where he or she works. Analysis was conducted using stepwise binary logistic regression. Four models were tested. Model I include variables regarding where care is given (nursing homes or home‐based care) and organization and contents of meetings with patients and relatives (Whether there is a standard agenda for meetings and how much time they usually devoted to various topics in these talks). Model II adds variables related to how EOL care is organized, indicated by the question of whether principles of primary nursing were applied and whether additional resources usually were allocated when patients were close to death. Model III adds variables characterizing nurses' personal background and model IV adds characteristics of the municipalities where the care is provided. Hosmer and *Lemeshow goodness* of fit was used to test the fit of the various models and *Nagelkerke pseudo R* ^*2*^ was used as estimate for explained variance. Binary coded variables included in the analysis are presented in appendix.

## 6 RESULTS

More than half of the nurses in EOL care had experienced that a dying patient's relatives found it difficult to accept the imminence of death (Table 1). A similar number reported that they have experienced disagreement among a patient's relatives concerning important decisions about life‐prolonging medical interventions. However, very few (4.3% & 3.8%, respectively) reported that this happens often.

**Table 1:** Percentage (frequencies) of nurses experiencing that relatives find it difficult to accept that the death is imminent disagreement among relatives concerning patient care (*N* = 399)

	Often	Sometimes	Seldom/never	No answer	Total (*N*)
Relatives find it difficult to accept that the death is imminent	4.3 (17)	51.1 (204)	44.2 (176)	0.5 (2)	100.0 (399)
Disagreement between relatives	3.8 (15)	49.9 (199)	44.6 (178)	1.8 (7)	100.0 (399)

Nurses working in home‐based care, more often than their colleagues in nursing homes do, experienced that a patient's family finds it difficult to accept that death is imminent (69.2% and 58.2%, respectively). Regarding the question of disagreements among relatives, however, no similar difference was reported (Table 2).

**Table 2:** Nurses experience of whether relatives find it difficult to accept that death is imminent and experience of disagreement between relatives according to where the care is given

	Care given at home (*N*) *n* %	Care given in nursing home (*N*) *n* %	Pearson's chi‐square
Difficult to accept that the death is imminent	(201) 139 69.2	(196) 114 58.2	0.02
Disagreement among relatives	(196) 110 56.9	(195) 111 56.1	0.87

Forty‐five percent (*N* = 181) of the nurses answered that they were sometimes themselves in doubt when questions regarding life prolongation were discussed. Forty‐seven percent (*N* = 187) reported that information to patients and their relatives were given in meetings with a preplanned agenda, while 33% (*N* = 133) answered that content of these meetings depended on what the patients or their relatives wanted to discusses. Twenty percent did not answer this question (Tables not shown). Table 3 shows which items they devoted most time to in these meetings. Sixty‐four percent said that they devoted a lot of time to “palliative care,” while only 18.5% devoted much time to “life‐prolonging medical interventions.”

**Table 3:** Percentage (frequencies) of nurses reporting “A lot,” “Some” or “A little” time devoted to specific issues in communication with patients' relatives (*N* = 399)

	A lot	Some	A little	No answer	Total (N)
Palliative care	64.7 (258)	31.1 (124)	3.0 (12)	1.3 (5)	100.0 (399)
Physical changes to be expected	32.8 (131)	59.4 (237)	5.8 (23)	2.0 (8)	100.0 (399)
Life‐prolonging medical interventions	18.5 (74)	46.9 (187)	28.1 (112)	6.6. (26)	100.0 (399)
Other medical questions	24.8 (99)	61.2 (244)	11.8 (47)	2.3 (9)	100.0 (399)

Sixty‐three percent (*N* = 251) of our respondents reported that additional nursing capacity is usually allocated when a patient enters the terminal phase. Thirty percent (*N* = 119) said that this was done only in situations where patients had special needs. Thirty percent (*N* = 112) reported that one of the nurses had a primary responsibility for the patient in this phase. Fifty‐eight percent (*N* = 321) reported that a defined group of nurses shared this obligation (Tables not shown).

Table 4 shows the result of a multivariate analysis of factors associated with the nurses' experiences of relatives' problems of acceptance when death is imminent. It shows that the probability of this is influenced by organizational and structural preconditions for care, characteristics of the process of communication and characteristics of the nurse. Nurses working in home‐based care more often reported that patients' relatives could find it difficult to accept it when death is imminent than their colleagues in nursing homes did. Further, nurses who devoted time to life prolongation in meetings with relatives and patients reported more often than others that the relatives found it difficult to accept that the patient was dying. Controlling for nursing organization, institutional or home‐based care, nurses' personal background, number of years in EOL care and the kind of municipality where the patient lives does not influence the strength of this association. Nurses who had doubts and felt insecure regarding questions of life prolongation reported more often than others experience that a patient's relatives may find it difficult to accept that death is imminent.

**Table 4:** Factors associated with nurses' experiences of difficulties for relatives that the death was imminent. Binary logistic regression: odds ratio, (95% confidence interval) *N* = 399

Variables in the models	I (OR, 95% CI)	II (OR, 95% CI)	III (OR, 95% CI)	IV (OR, 95% CI)
Where care is given (home‐based = 1, nursing home = 0)	0.53 (0.30–0.85)	0.46 (0.27–0.80)	0.52 (0.28–0.94)	0.53 (0.28–0.95)
Meetings have a standard agenda (yes = 1)	0.74 (0.45–1.21)	0.75 (0.45–1.25)	0.71 (0.42–1.22)	0.75 (0.43–1.30)
Talk about physical changes (yes = 1)	0.86 (0.50–1.49)	0.81 (0.46–1.42)	0.82 (0.45–1.48)	0.76 (0.41–1.39)
Talk about palliation (yes = 1)	0.95 (0.53–1.75)	1.01 (0.55–1.85)	1.04 (0.55–1.99)	1.05 (0.54–2.06)
Talk about life prolongation (yes = 1)	2.15 (1.27–3.65)	1.95 (1.13–3.53)	2.01 (1.14–3.53)	1.97 (1.08–3.60)
Talk about other medical questions (yes = 1)	0.82 (0.46–1.47)	0.83 (0.45–1.51)	0.88 (0.47–1.66)	0.86 (0.45–1.64)
Have a primary nurse (yes = 1)		0.63 (0.36–1.10)	0.60 (0.33–1.01)	0.68 (0.36–1.26)
Additional nurse capacity available (yes = 1)		1.06 (0.63–1.79)	1.12 (0.64–1.94)	1.09 (0.62–1.91)
Nurse often feel insecure (yes = 1)			3.10 (1.78–5.40)	3.19 (1.80–5.64)
More than 10 years in geriatric practice (yes = 1)			0.74 (0.38–1.44)	0.82 (0.42–1.60)
Small municipalities (yes = 1)				1.13 (0.60–2.11)
Long travel time to hospital (yes = 1)				1.15 (0.59–2.24)
Hosmer and Lemeshow goodness of fit (significance)	0.64	0.13	0.04	0.13
Nagelkerke pseudo *R* ^2^	0.08	0.09	0.18	0.19
Constant	2.13	2.66	1.81	0.78

Table 5 gives the results of a multivariable analysis of factors influencing the probability for a nurse to experience disagreements between relatives of dying patients regarding whether life‐prolonging treatment is in accordance with a patient's wishes. Nurses who devoted substantial time to the question of life prolongation reported more often that they experienced contradictory viewpoints between relatives on this matter. This seems to hold irrespective of nursing organization, type of care, nurses' personal background or characteristics of the municipality where the patient live. When additional nursing resources were allocated when patients enter the terminal phase, nurses experienced fewer conflicts among relatives. However, this association vanishes when controlling for type of municipality. As was the case regarding the problem of relatives' “denial” (Table 4), the incidence of conflicts between relatives seems dependent on what the nurses' think and feel. If nurses have doubts and feel insecurity regarding what is best for the patient, the probability of conflict between the patient's relatives is high. According to our data, how long the nurses have worked in this kind of care seems unimportant.

**Table 5:** Factors associated with nurses' experiences of disagreements between relatives. Binary logistic regression: odds ratio, (95% confidence interval) *N* = 399

Variables in the models	I (OR, 95% CI)	II (OR, 95% CI)	III (OR, 95% CI)	IV (OR 95% CI)
Where care is given (home‐based = 1, nursing home = 0)	0.84 (0.52–3.18)	0.90 (0.54–1.01)	1.10 (0.62–1.94)	1.08 (0.60–1.95)
Meetings have a standard agenda (yes = 1)	1.06 (0.66–1.70)	0.98 (0.60–1.59)	0.99 (0.60–1.66)	0.96 (0.57–1.66)
Talk about physical changes (yes = 1)	1.15 (0.70–1.97)	1.14 (0.66–1.99)	1.17 (0.65–2.09)	1.08 (0.59–1.96)
Talk about palliation (yes = 1)	1.17 (0.66–2.07)	1.23 (0.69–2.21)	1.39 (0.75–2.60)	1.35 (0.71–2.58)
Talk about life prolongation (yes = 1)	2.19 (1.30–3.68)	2.26 (1.32–3.88)	2.47 (1.40–4.37)	2.76 (1.50–5.09)
Talk about other medical questions (yes = 1)	1.07 (0.61–1.90)	1.17 (0.65–2.10)	1.25 (0.67–2.33)	1.22 (0.64–2.30)
Have a primary nurse (yes = 1)		1.39 (0.80–2.39)	1.36 (0.75–2.45)	1.45 (0.77–2.69)
Additional nurse capacity available (yes = 1)		0.57 (0.35–0.98)	0.58 (0.33–0.99)	0.59 (0.34–1.04)
Nurse feels often insecure (yes = 1)			2.71 (1.59–4.62)	2.85 (1.65–4.95)
More than 10 years in geriatric practice (yes = 1)			0.62 (0.33–1.17)	0.66 (0.36–1.28)
Small municipalities (yes = 1)				0.52 (0.28–0.97)
Long travel time to hospital (yes = 1)				1.21 (0.65–2.26)
Hosmer and Lemeshow goodness of fit (significance)	0.68	0.68	0.36	0.918
Nagelkerke pseudo *R* ^2^	0.05	0.07	0.15	0.17
Constant	0.69	0.86	0.59	0.78

## 7 DISCUSSION

One of the interviewees, when preparing the questionnaire, explained why she considered work with dying patients worthwhile: “*It can be beautiful when the process is good*.” The process of losing a loved one, however, painful it may be, can give relatives a sense of relief, if relatives feel that they have been able to cope with the situation and make the last days together with their loved ones meaningful (Andershed, 2006). Our study indicates, however, that such an outcome is not always obtainable. Main findings were as follows:

- Imminence of death seemed easier to accept by the patient's relatives of patients dying in nursing homes than did relatives of patients in home‐based care.
- Nurses in smaller municipalities experienced fewer conflicts between relatives acting as proxy decision makers for patients unable to consent than did nurses in larger municipalities.
- Nurses who themselves felt doubt regarding the dilemmas of life extension for dying patients more often than others experienced that a patient's relative had problems to accept that death was close and that relatives were divided in opinion when they were asked to take decisions on behalf of a patient.

### 7.1 Imminence of death more easily accepted in nursing homes than in home‐based care

Accepting imminence of death seemed easier when patients were cared for in nursing homes, compared with home‐based care. One possible explanation for this may be that patients in nursing homes and home‐based care have very different experiences regarding dealing with death and dying. In a Norwegian nursing home, average length of stay is 2.9 years (Brevik, 2008). During their stay, most nursing home patients experience several deaths of their co‐habitants. Due to this, both patients and relatives will be better prepared for death and may find it easier to accept demise. Another possible explanation could be differences in characteristics of patients being cared for in nursing homes and at home, respectively. A higher probability of “denial” of imminent death in home‐based care, then, may be related to the characteristics of patients and the situation of their relatives. Another contributing factor may be differences regarding general working conditions for end‐of‐life care in nursing homes and patients' own homes, respectively. In their evaluation of palliative care in Norway, Melby and team found that nursing personnel in nursing homes worked more systematically to secure high quality in EOL care and described better conditions for their work with patients in need of palliative care than did their colleagues in home‐based care (Melby et al., 2016). As pointed out in the introduction, clinical experience of healthcare personnel and that collegial support at nursing homes is much stronger than in home‐based care setting, where nurses more often operate alone. The nursing home setting may enable better support and build trust among relatives. However, in home‐based care, nurses operating alone may find it more difficult to gain confidence and trust among relatives.

An important element in EOL care is the quality of collaboration between nurses and physicians. In home‐based care, nurses are dependent on collaboration with the patient's GP, who works on a fee‐for service‐basis. A study of collaborative patterns between GPs and other healthcare workers in Norwegian primary health care by Steihaug, Paulsen and Melby, however, concludes that such collaboration is hampered by organizational, economical and other structural conditions inbuilt in the primary healthcare organization itself (Steihaug, Paulsen, & Melby, 2017). It seems reasonable to believe that preconditions for nurse/physician‐collaboration will be better in nursing homes, served by nursing home physicians with a permanent employment on a part‐time basis. It seems obvious, then, that EOL care in patients' own homes may be a more demanding situation regarding the interplay between the patient's, their relatives and health personnel serving them, compared with care of dying patients in nursing homes.

In a recent Norwegian governmental official report, undertaken by an expert committee assigned by Ministry of Health and Care Services, comprehensive recommendations for improving the quality and coordination of EOL patient trajectories are given (Official Norwegian Report, 2017). The committee document the wide‐ranging challenges in the expanded field of palliative care. To strengthen the palliative EOL patient trajectories in Norway, the committee underscore that the strengthening of competence, organization and education is crucial (Official Norwegian Report, 2017). In this setting, special attention should be given to home‐based care. Especially, more training is needed to prepare nurses for EOL care in a home‐based setting.

### 7.2 Fewer conflicts between patient's relatives in smaller municipalities

Type of municipality made a difference regarding nurses' experiences of how death and dying was perceived by patients' relatives. Nurses working in smaller municipalities experienced fewer disagreements among relatives acting as proxy decision makers for a patient. However, this finding was associated with a tendency to allocate more personnel when a patient entered terminal phase. This indicates that many smaller municipalities have a flexibility to make temporary adjustments of service level when needed, which seems to be uncommon in larger municipalities. This may be a reflection of general economic differences regarding municipal spending on long‐term care. A study of variations between Norwegian municipalities regarding resources allocated to long‐term care shows that small and remote municipalities usually spend more money per capita on long‐term care, have more nursing home beds and produce home services in patients' own homes, in comparison with larger municipalities (Husebye & Paulsen, 2009). It seems reasonable to infer that more resources—giving more flexibility to allocate additional nursing personnel to EOL care when needed—enable nurses to spend more time with the patient and the patient's relatives. Thus, nurses can help relatives develop a better and more consistent understanding of the patient's interests, when they make decision on his/her behalf. Organization and staffing of care when death is close, then, need careful consideration. Our study indicates that to mobilize additional nursing capacity when a patient enters terminal phase strengthens relatives' ability to support the patient and thereby heighten the quality of care during patient's last days.

### 7.3 Associations between nursing practice and experienced difficulties in accepting that death is imminent

Our study demonstrated a strong association between experienced difficulties in the group of relatives and variations in characteristics of the nursing practice itself. Nurses who devoted more time than others to talk about life‐prolonging medical treatment and nurses who themselves were uncertain about whether life prolongation was in the patient's best interests reported more often than others that relatives found it difficult to accept that death was imminent and that disagreements between relatives occurred when they had to act as proxy decision makers on behalf of the patient. Similar associations were not found for other variables regarding the communication between health personnel, patients and their relatives. The same goes for variables characterizing nurses' professional background, that is, years in geriatric practice.

The question of maintenance or withdrawal of life‐prolonging medical treatment is consequential and ambiguous. On the one hand, it may provide the possibility of postponement of death and additional time together with a loved one. On the other hand, life‐prolonging interventions may involve more suffering for the dying person, disturb the possibilities for being together in a calm and confident atmosphere and may even increase the risk of a contradictory outcome (i.e., the patient may die sooner than would otherwise be expected). Confrontation with these types of questions may be a heavy load for the relatives of patients who are unable to express their wishes (Andershed, 2006; Dreyer et al., 2009). In her interviews with relatives of recently deceased family members, Dreyer (2009) found that many felt confronted with a responsibility they were not prepared for. In this situation, then, a clear advice from a nurse could be a relief for a dying patient and an exhausted family. In this perspective, not being able to give clear advices in this very difficult situation could be considered a lack of professionalism and an obstacle for good care. One of the major critiques against the use of LCP, formulated in the report by the independent investigation in Great Britain, was the unqualified broad application of the pathway, in spite of the difficulties to establish when patients were dying. Further, lacking and poor communication between healthcare personnel and relatives resulted in uncertainty about withdrawing and withholding treatment and the application of palliative care (Melby et al., 2016, p. 28). An adequate awareness among healthcare personnel and clinical competence is crucial when applying an EOL care pathway.

From another point of view, however, not being able or willing to give clear and authoritative advices in this very difficult situation may be rooted in the nurse's attitudes and ethical considerations towards the ambiguity in the death process itself. Several studies confirm that patients' and relatives' awareness of death varies and fluctuates (Borneman et al., 2014; Richards et al., 2013; Small & Gott, 2012; Timmermans, 1994). Timmermans criticized, nuanced and modified Glaser and Strauss's theory in an article published in 1994. He underscored that acceptance of death and open awareness cannot be imposed on all patients and relatives. Further, clinging to unrealistic hopes of recovery and denial of the imminence of death can be a mental and emotional strategy for coping with the situation (Timmermans, 1994).

Gina Copp and David Field concluded in their study of the interplay between dying patients, their family and nursing personnel that fluctuation between open awareness and denial of death should be seen as a coping strategy, very important for both the patient and the family; “It appears that dying individuals use denial and acceptance alternatively as means of coping. By providing a “safety net,” albeit temporarily, it would appear that denial serves to preserve self‐esteem, maintain existing relations and prevent disintegration at certain periods of the dying process” (Copp & Field, 2002, p. 126). A similar conclusion was made by Borneman and colleagues, when commenting a patient trajectory “…using “denial” to give her hope was not an act of denying death, but rather an act of affirming life and what was important to her.” (Borneman et al., 2014). In this fluctuating situation, there may even be differences in interests and wishes between the patients and their relatives. In a recent survey about EOL care issues undertaken by the Norwegian nursing Union' journal *Sykepleien* (Hofstad, 2017), 1141 nurses responded. Out of these nurses, 39% worked in healthcare institution in the local municipalities in Norway. In this survey, 68% of the nurses in the municipalities reported that they had experienced that relatives' wishes about continued life‐prolonging treatment or withdrawal/withholding treatment had been given more weight than patients' own wishes (Hofstad, 2017, pp. 32–34). Hofstad's data underscore nurses' high degree of compliance with relatives' wishes at the terminal stage in this context. One reason for this high compliance could be nurses' strife to avoid conflicts.

Nurses caring for patients during their last days work in a setting characterized by tension between hope and awareness of death. Our findings may indicate that some nurses, more than others, sense and accept fluctuations between hope and resignation when helping patients and their relatives to cope, although this may complicate EOL care. Maybe, then, we may identify two different groups of nurses engaged in end‐of‐life care. The first one consists of nurses willing to give authoritative advices to patients and their relatives regarding the most difficult questions in end‐of‐life care, based on their professional considerations of what will be the best for the patient and the relatives. The other group consists of nurses who orientate themselves more by what they believe are the patient's wishes, accepting the importance of fluctuations between unrealistic hopes and acceptance of the inescapable. Our data show that this is not influenced by the number of years of practice in end‐of‐life care, but is probably rooted in some deeper and basic consideration regarding the needs of a patient facing death.

## 8 CONCLUSION

With a decentralization of EOL care, in the wake of the *Coordination Reform* and with a growing number of older people in Norwegian population, more people will be cared for in municipal nursing homes and home‐based care and a growing number will spend their last days at home. EOL care in patients' own homes is a more demanding situation than care in nursing homes regarding interplay between patients, relatives and nurses. Further, contemporary palliative care in Norway includes a wide spectrum of diseases, in addition to a focus on oncology, adding to the challenges in EOL care. For instance, difficulties with predicting imminent death of patients with dementia or cognitive impairment (Melby et al., 2016, p. 133). Open awareness, advocated by Glaser and Strauss and invoked by contemporary palliative care ideology, is a demanding principle in the Norwegian setting. If this principle is to be applied fully in long‐term EOL care in Norway, there will be a need for allocation of extensive resources in the form of enhanced coordination of patient trajectories, palliative care support teams, supervision and properly trained healthcare personnel.

Our data showed that there are consequential discrepancies between different groups of nurses' regarding their thinking about what was best for the patient and how to understand a dying patient's wishes. More research is needed to widen our knowledge about the interplay between the patient, relatives and the nurses working with dying patients in different caring levels in Norway.

## 9 STRENGHTS AND LIMITATIONS

The strength of this study was the sampling criteria for experienced nurses. EOL care can be learned through experience, which was the most important qualifications for answering our survey and conclusions to be drawn from them. The survey was stratified according to municipality type. This was done to facilitate analysis of organizational differences associated with small and remote municipalities compared with those that are larger and more central. However, the relatively low response rate must be considered.

## APPENDIX 1 Variables in Tables 4 and 5.

### 1.1

Variable type: dummy variables, values 1 and 0.

**Table:**

Variable name	Values	%	*N*
Difficult to accept that the death is imminent	1 = Relatives often or sometimes find it difficult to accept that death is imminent 0 = Relatives never or seldom find it difficult to accept that death is imminent	0.68	397
Disagreement among relatives	1 = Relatives often or sometimes disagree 0 = Relatives never or seldom disagree	0.57	391
Meetings have a standard agenda	1 = Meetings have a standard agenda 0 = Agenda dependent on relatives wishes	0.58	320
Talk a lot about physical changes	1 = Much time devoted to physical changes 0 = Do not devote much time to physical changes	0.34	391
Talk a lot about palliation	1 = Much time devoted to palliation 0 = Do not devote much time to palliation	0.65	394
Talk about life prolongation	1 = Much time devoted to life prolongation 0 = Do not devote much time to life prolongation	0.68	382
Talk a lot about other medical questions	1 = Much time is devoted to other medical questions 0 = Do not devote much time to other medical questions	0.25	391
Patients have a primary nurse	1 = One dedicated nurse has a primary responsibility for the patient 0 = Several nurses share responsibility	0.31	392
Additional nursing capacity is allocated	1 = Additional capacity is usually allocated when a patient is dying 0 = Additional nursing capacity usually not allocated	0.64	395
Home‐based care	1 = Nurse works in home‐based care 0 = Nurse works in a nursing home	0.50	399
Nurse often feels unsecure	1 = Nurse herself frequently feels insecure regarding life prolongation 0 = Nurse herself very seldom feels insecure	0.46	393
More than 10 years in geriatric care	1 = Nurse has worked more than 10 years with care of elderlies 0 = Nurse has worked less than 10 years with care of elderlies	0.73	387
Rural municipality	1 = Nurse works in a municipality with less than 10,000 inhabitants 0 = Nurse works in a municipality with more than 10,000 inhabitants	0.64	393
Travel distance to hospital	1 = Travel to hospital takes more than 1 hr 0 = Travel to hospital takes less than 1 hr	0.28	390
